# Dynamic Switch Between Two Adhesion Phenotypes in Colorectal Cancer Cells

**DOI:** 10.1007/s12195-013-0313-8

**Published:** 2013-11-14

**Authors:** Yue Geng, Siddarth Chandrasekaran, Sivaprakash Agastin, Jiahe Li, Michael R. King

**Affiliations:** Department of Biomedical Engineering, Cornell University, Ithaca, NY 14853 USA

**Keywords:** Cell adhesion, E-selectin, *β*-Catenin, E-cadherin, Metastasis

## Abstract

The hematogenous metastatic cascade is mediated by the interaction of cancer cells and the endothelial cell lining of blood vessels. In this work, we examine the colon cancer cell line COLO 205, which grows simultaneously in both adherent and suspended states in culture and can serve as a good model for studying tumor heterogeneity. The two subpopulations of cells have different molecular characteristics despite being from the same parent cell line. We found that the ratio of adherent to suspended cells in culture is maintained at 7:3 (equilibrium ratio). The ratio was maintained even when we separate the two populations and culture them separately. After 8 h in culture the equilibrium was achieved only from either adherent or suspended population. The adherent cells were found to express less E-selectin binding glycans and demonstrated significantly weaker interaction with E-selectin under flow than the suspended cells. Manipulation of the epithelial–mesenchymal transition (EMT) markers *β*-catenin and E-cadherin expression, either by siRNA knockdown of *β*-catenin or incubation with E-cadherin antibody-coated microbeads, shifted the ratio of adherent to suspended cells to 9:1. Interestingly, human plasma supplemented media shifted the ratio of adherent to suspended cells in the opposite direction to 1:9, favoring the suspended state. The dynamic COLO 205 population switch presents unique differential phenotypes of their subpopulations and could serve as a good model for studying cell heterogeneity and the EMT process *in vitro*.

## Introduction

Colorectal cancer is one of the most frequent human tumors, which can metastasize to liver, lung, and peritoneum. Significant intratumoral genetic heterogeneity has been demonstrated in advanced colorectal carcinoma, indicating the importance to re-evaluate the use of genetic markers for prognosis.^2^ Genetic heterogeneity is known to play a key role in determining which cells from the primary tumor ultimately metastasize. The heterogeneity dictated by reduction or loss of E-cadherin that mediates homotypic cell–cell adhesion has been shown to promote the progression of several carcinomas including colorectal cancers.^15^,^16^,^20^,^22^ This reduced cell–cell adhesiveness permits cells to deviate from normal cell growth patterns, resulting in the destruction of histological structures. As an important member of the cadherin family, the cytoplasmic domain of the Ca^2+^ dependent transmembrane glycoprotein E-cadherin regulates the structural and signaling activities required for adhesion through interactions with *β*-catenin, *α*-catenin and plakoglobin (*γ*-catenin).^29^ Wnt signal mediated tyrosine phosphorylation of *β*-catenin has been established as an important regulatory mechanism behind cytoplasmic protein stabilization as the phosphorylated protein has less affinity to both the APC/GSK-3/Axin complex and E-cadherin.^6^ The loss of E-cadherin mediated cell–cell adhesion and phosphorylation of *β*-catenin has been shown to play an important role in the metastasis of colorectal cancer.^4^

Metastasis is a complex and highly organized process that involves a series of distinct steps.^19^ The formation of metastases by invasive transformed cells accounts for 90% of all deaths in cancer patients.^31^ To form secondary tumors, cancer cells must invade the surrounding tissue and enter either the bloodstream or the lymphatic system. Similar to the leukocyte adhesion cascade which is initiated *via* cell tethering and rolling through selectin mediated interactions, cancer cells displaying selectin ligands are potentially involved in a series of events that eventually lead to metastasis, i.e. the metastatic cascade.^9^,^14^,^17^,^21^ Following intravasation, selectins can interact with a variety of *O*-glycosylated ligands and, in turn, mediate the transient adhesion of cells to the endothelium. These receptor–ligand interactions represent the first contact between cancer cells and the endothelial wall, which facilitates tethering and rolling events.^10^ Sialic acids are terminal monosaccharides attached to cell surface glycoconjugates and play important roles in many physiological and pathological processes.^11^ Over a decade ago, researchers found that tumor cells overexpress sialyl Lewis x (sLe^x^) or sialyl lewis a (sLe^a^) on their surface glycoproteins and/or glycosphingolipids in lung, colon, gastric, and pancreatic carcinomas.^3^ sLe^x^-bearing human colon adenocarcinoma cell lines including COLO 205 and LS174T have been shown to undergo extensive adhesive interactions with E- and P-selectin under flow conditions.^1^,^12^ The human colon carcinoma cell line COLO 205 was established from the ascetic fluid of a male patient with poorly differentiated colorectal carcinoma by Semple *et al.* 27 Interestingly, this cell line has a unique morphological character in that it grows simultaneously in both adherent and suspended states in culture. In this work, we observed that when separated, both suspended and adherent populations of COLO 205 generate their counterpart populations and re-distribute so that the ratio of adherent cells to suspended cells in culture is always 7:3. This is referred to as the “equilibrium ratio” in the rest of the paper. In this study, we further examined the kinetics of this phenomenon and investigated the tumor heterogeneity between the COLO 205 adherent and suspended populations and biochemical factors that modulate this equilibrium ratio.

## Materials and Methods

### Cell Line and Cell Culture

The COLO 205 cell line was obtained from ATCC (American Type Culture Collection, Manassas, VA). Gibco^®^ RPMI media (Life Technologies, Grand Island, NY), supplemented with 10% fetal bovine serum (Life Technologies, Grand Island, NY), 100 IU/mL penicillin and 10 *μ*g/mL streptomycin (Life Technologies, Grand Island, NY) was used as growth media. Cells were cultured in BD Falcon™ 75 cm^2^ cell culture flasks at 37 °C in an incubator supplied with 5% CO_2_. To collect the adherent COLO 205 cells, enzyme free dissociation buffer (Life Technologies, Grand Island, NY) was added after removing the suspended population. 90% cell viability was confirmed with trypan blue dye exclusion using a hemocytometer.

### Cell Population Assay

Adherent and suspended populations of COLO 205 cells were separated and deposited in 6-well plates. At time points 0, 0.5, 1, 2, 4, 6, and 8 h, cells were collected and counted as described above. In separate experiments, adherent and suspended COLO 205 cells were separated and labeled with CellTracker Green and Orange (Life Technologies, Grand Island, NY), respectively. The adherent green cells were then re-plated at 0.2 million cells per well in a 12-well plate and allowed to reach an equilibrium adherent:suspended ratio for 8 h. Likewise, the suspended orange cells were re-plated at the same concentration and equilibrated for 8 h. The suspended and adherent populations for both the green and orange cultures were separated again. Finally, the orange suspended cells were deposited into the wells containing only the green adherent layer. The numbers of orange and green cells in the suspended and adherent layers were counted from 500 *μ*L cell solutions of each population using a flow cytometer every 2 h for 10 h, to assess the rate of phenotype “flip-flop”.

### Reverse Transcription

Total RNA from COLO 205 suspended and adherent cells was prepared and purified separately using RNeasy Plus Mini kit (Qiagen). The 40 *μ*L reverse transcription reaction system includes 10 *μ*g of total RNA, 1 *μ*L of M-MuLV Reverse Transcriptase (New England Biolabs), 0.5 *μ*L of RNase Inhibitor (New England Biolabs), 1 *μ*L of Random Primers (Invitrogen), 2.5 *μ*L of dNTP Mix (New England Biolabs) and 4 *μ*L of MuLV Reverse Transcriptase reaction buffer (New England Biolabs). The reaction mixture was incubated inside the RT-PCR (Bio-Rad) instrument at 42 °C for 1 h, followed by an inactivation step at 95 °C for 10 min.

### Real-Time Quantitative PCR (qPCR)

10 ng of cDNA produced by the reverse transcription of total RNA was used in each qPCR reaction. Also included in the 20 *μ*L qPCR reaction system were 10 *μ*L iQ™ SYBR Supermix (Bio-Rad), 1 *μ*L of 2 *μ*M forward primer and 1 *μ*L of 2 *μ*M reverse primer and nuclease free water.

### Primer for *β*-Catenin qPCR (Product Size: 166 bp)

5′-GAAACGGCTTTCAGTTGAGC-3′ (forward)

5′-CTGGCCATATCCACCAGAGT-3′ (reverse)

### Primer for GAPDH qPCR (Product Size: 170 bp)

5′-AGAGCACAAGAGGAAGAGAGAGAC-3′ (forward)

5′-AGCACAGGGTACTTTATTGATGGT-3′ (reverse)

qPCR reactions were carried out in 96-well real-time PCR plates (Bio-Rad) using a Bio-Rad MyIQ Real-time PCR detection system. The qPCR reaction included 5 min at 95 °C to activate the polymerase and 50 PCR cycles (uncoupling step at 95 °C for 20 s followed by annealing step at 59 °C for 20 s and elongation step at 72 °C for 30 s), followed by a melting temperature analysis to test for any nonspecific amplification. All the qPCR reactions were performed in triplicate. The expression level of *β*-catenin gene in each cell population was normalized to the expression level of the standard gene GAPDH.

### siRNA Transfection

*β*-Catenin siRNA was purchased from Applied Biosystems (Silencer^®^ pre-designed & validated siRNA, ID: s436). Lipofectamine™ RNAiMAX (Invitrogen) reagent was used to transfect the COLO 205 cells with *β*-catenin siRNA as described by the manufacturer.

### E-cadherin Antibody Coated Bead Incubation

Protein A coated polystyrene beads (500 *μ*L, 1% w/v, Spherotech, Lake Forest, IL) were first incubated with 2 mL of 50 *μ*g/mL mouse anti-human E-cadherin monoclonal antibodies (Santa Cruz Biotechnology, Santa Cruz, CA) on ice for 45 min. Conjugated beads were washed once with 1 mL PBS and then resuspended with 500 *μ*L of fresh media. 250 *μ*L of bead-containing media was added to COLO 205 cells cultured in 6-well plates and incubated for 2 h at 37 °C before cell counting and mRNA analysis.

### Flow Cytometry

Adherent and suspended populations of COLO 205 cells were separated, washed with 1× DPBS, and resuspended in 1× DPBS with 1% BSA to a final concentration of 20,0000–30,0000 cells in each sample. Antibodies or appropriate isotype controls were added to cell suspensions and incubated over ice for 45 min. Following the incubations, the cells were washed three times with 1 mL of 1× DPBS to remove any unbound antibody. Flow cytometry samples were analyzed using an Accuri C6 flow cytometer (Accuri Cytometers Inc., Ann Arbor, Michigan, USA) and plots were created using the FCS Express package.

### Proteome Profiler Assay

Human Phospho-kinase Antibody array kit (R&D systems) was used to analyze the phosphorylation profiles of several key kinases and their protein substrates in both the adherent and suspended COLO 205 cells. Adherent and suspended cells were separated, washed in ice cold PBS buffer, and pelleted before adding the lysis buffer provided with the array kit. Total protein concentrations were determined by Bradford colorimetric assay (Bio-Rad). The nitrocellulose membranes provided with the kit were processed and developed as per the manufacturer’s instructions.

### Immunoblotting

Whole cell lysate from both subpopulations was freshly made using RIPA lysis and extraction buffer. The lysate protein concentrations were measured using Bradford assay (BioRad). SDS-PAGE was performed using 7.5% polyacrylamide gels and proteins were transferred to a nitrocellulose membrane, incubated with primary antibodies against *β*-catenin (Biolegend) and *β*-actin (Santa Cruz Biotechnology) overnight at 4 °C. Goat anti-rabbit IgG-HRP (Santa Cruz Biotechnology) and rabbit anti-mouse IgG-HRP (Abcam) were used as secondary antibodies.

### Cell Rolling Assay

Microrenathane tubing with 300 *μ*m internal diameter (Braintree Scientific) was cut to a length of 50 cm, functionalized with Protein G (10 *μ*g/mL) and Fc chimera E-selectin (20 *μ*g/mL, R&D), and blocked with 5% BSA or milk (Sigma). Functionalized microtubes were then secured to the stage of an Olympus IX81 motorized inverted microscope (Olympus America, Melville, NY). A CCD camera (model no: KP-M1AN, Hitachi, Tokyo, Japan) and DVD recorder (model no: DVD-1000MD, Sony Electronics) were used to record experiments for offline analysis. Adherent and suspended COLO 205 cells were separated and suspended in flow buffer at 1 × 10^6^ cells/mL and perfused through protein coated microtubes using a syringe pump (KDS 230, IITC Life Science, Woodland Hills, CA) at a wall shear stress of 1.0 dyn/cm^2^.

### qPCR Profiling of EMT-Associated Genes

RNA was extracted from adherent and suspended cells by TRIZOL method. First strand cDNA was synthesized using the Invitrogen first strand cDNA synthesis kit. Qiagen EMT PCR array including qPCR primers for signature genes during EMT was performed in the Biorad iQ qPCR machine to compare gene expression exhibited by the two populations using the 2^−Δ*Ct*^ method. Gene expression was normalized to the housekeeping genes GAPDH and *β*-actin.

### Plasma Isolation and Treatment

Whole peripheral blood was drawn from informed consenting healthy donors by venipuncture into BD Vacutainer tubes. Collected whole blood was centrifuged for 25 min at 500 rpm. The plasma layer on top was carefully removed without disturbing the interface and passed through a sterile 0.2 *μ*m filter. 50% of the isolated plasma supplemented culture media was used to culture COLO 205 cells at 2 × 10^5^ cells/well in 6-well plates prior to experiments.

## Results

### Separation of Adherent and Suspended Subpopulations Resulted in the Re-establishment of the Equilibrium Ratio

**Figure 1:**
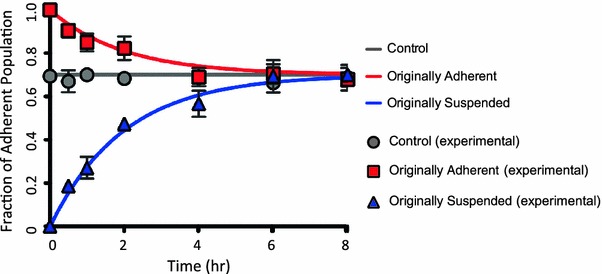
COLO 205 adherent and suspended population reformation and stabilization. Adherent and suspended cells were separated and re-plated in fresh RPMI media (data referred to as ‘originally adherent’ and ‘originally suspended’). The originally adherent and suspended culture flasks were monitored over 8 h by performing cell count at 0.5, 1, and 2 h intervals and the fraction of adherent cells in all conditions was plotted against time. A simple ordinary differential equation (ODE) model was derived for the adherent fraction as a population balance on adherent and suspended cells. Solutions to the ODE model were plotted for the best-fit model parameters of *k* = 0.5 h^-1^ and *A*_eq_ = 0.7

When cultured in monolayers, COLO 205 cells grow simultaneously in both adherent and suspended states. While the adherent COLO 205 cells grow in small aggregates (2-dimensional islands), the suspended population grows as individual cells. To separate the two populations, suspended cells were collected and plated in a new culture flask while fresh media was added to the original flask containing the adherent population. Cell counting and viability assay were performed using trypan blue dye and a hemocytometer at various time points including 0, 0.5, 1, 2, 4, 6, and 8 h. Interestingly, each flask initiated with either all adherent or all suspended cells was observed to re-establish an “equilibrium ratio” of 70% adherent cells to 30% suspended cells within 8 h, as shown in Fig. 1. A simple mathematical model was formulated to describe the kinetics for the adherent (*A*) and suspended (*S*) cells to re-establish the equilibrium ratio:

1 $$ \frac{dA}{dt} = - k_{1} A + k_{2} S $$

$$ A + S = N({\text{Total}}\;{\text{number}}\;{\text{of}}\;{\text{cells)}} $$

$$ {\text{at}}\;{\text{equilibrium}},\;\% \;{\text{adherent cells}} = A_{\text{eq}} $$

$$ \frac{dA}{dt} = 0 $$

2 $$ 0 = - \left( {k_{1} + k_{2} } \right)A_{\text{eq}} + k_{2} N. $$

The above differential equation gives the general solution,

$$ A = c_{1} e^{{ - \left( {k_{1} + k_{2} } \right)t}} + c_{2} $$

with initial conditions

$$ t = 0,\;A = A_{0},\;{\text{where}}\;A_{0}\;{\text{is}}\;{\text{the}}\;{\text{initial}}\;{\text{number}}\;{\text{of}}\;{\text{cells}}\;{\text{in}}\;{\text{the}}\;{\text{adherent}}\;{\text{state}}) $$

This yields the solution

3 $$ A = \left( {A_{0} - A_{\text{eq}} } \right)e^{{ - \left( {k_{1} + k_{2} } \right)t}} + A_{\text{eq}} . $$

The best-fit solution to this simple model (Eq. 3) was compared with the experimental data in Fig. 1, and shows excellent agreement, implying that the phenotypic switch can be approximated with first-order kinetics. The constants *k*_1_ and *k*_2_ of the first order kinetics solution can be attributed to cell proliferation and cells switching from adherent state to the suspended state.

### Dynamic Switching Between the Suspended and Adherent Cells Occurs Rapidly and Reaches an Equilibrium Over Time

**Figure 2:**
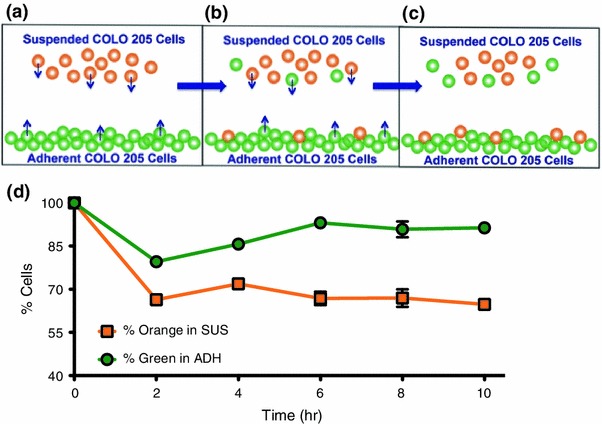
(a–c) Dynamic switch between the adherent and suspended COLO 205 populations. (d) Percentage of orange CellTracker dye labeled cells in the suspended layer (SUS) and green CellTracker dye labeled cells in the adherent layer (ADH) over 10 h

The adherent and suspended cells were separated and labeled with CellTracker green and CellTracker orange dyes, respectively. To determine whether a dynamic switch between the two states maintained the existence of two subpopulations, the labeled cells were plated separately at the same seeding density. After they reached the previously observed equilibrium ratio (time to reach equilibrium ratio was determined by previous experiments to be 8 h), the suspended layer from the originally suspended population (in orange) was added to the adherent layer of the originally adherent type (in green) (Fig. 2a). After 2 h, the cells in suspension were removed and analyzed using flow cytometry. The results revealed that 70% of cells in the suspended population were positive for CellTracker orange (conversely, 30% of cells were positive for CellTracker green, which were originally adherent) (Fig. 2d). We also collected the adherent cells and analyzed these cells for CellTracker green labeling. Only 85% of cells in the adherent population were positive for CellTracker green (conversely, 15% of cells were positive for CellTracker orange, which were originally suspended) (Fig. 2d). At *t* = 4 h there was an increase in the percentage of orange and green cells in the suspended and adherent populations respectively, indicating that some cells that had switched from one state to the other at *t* = 2 h switched back again. These results indicate that there is a dynamic switch between cells in the two subpopulations (Figs. 2b and 2c).

### Phosphorylated Kinases are Upregulated in the Suspended Population

**Figure 3:**
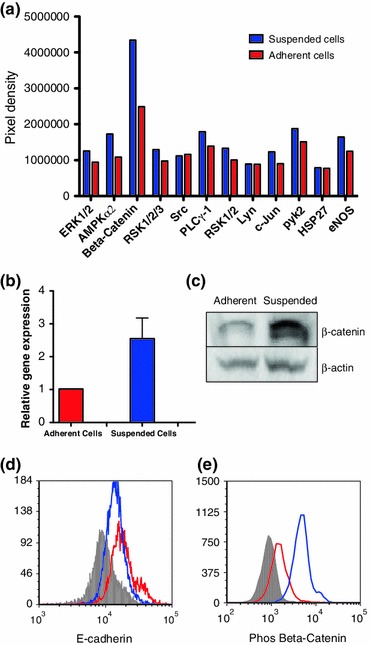
(a) Phosphorylation levels of COLO 205 adherent and suspended cells. (b) Relative *β*-catenin gene expression comparison between the adherent and suspended populations. Suspended cells have an average of 2.5-fold higher *β*-catenin expression. (c) Immunoblotting of *β*-catenin protein expression in the two populations. (d) and (e) Flow cytometry plots of anti-E-cadherin and anti-phosphorylated *β*-catenin antibody labeling. Isotypes, suspended population, and adherent population were colored in grey, blue, and red respectively

Relative phosphorylation levels of 12 key kinases and their protein substrates in both adherent and suspended COLO 205 cells were measured. As depicted in Fig. 3a, 9 out of 12 key kinases were found to be expressed at relatively elevated levels in the suspended COLO 205 cells compared to their adherent counterparts, among which the phosphorylation of *β*-catenin showed the greatest increase. The overall increase in the kinase phosphorylation activity in suspended cells can be explained by their significantly higher *β*-catenin gene expression (Fig. 3b). The increased phosphorylation of *β*-catenin protein observed in the suspended cells is also expected to reduce their binding affinity to both E-cadherin and APC, causing the suspended cells to detach and form individual cells in suspension. Increased kinase phosphorylation activity has also been recently reported to inhibit integrin mediated cell–ECM interactions.^28^

### Suspended Cells Show Increased Expression of *β*-Catenin and Decreased Expression of E-cadherin

Immunoblotting results, shown in Fig. 3c, indicated that the total (surface+intracellular) *β*-catenin protein expression was elevated in the suspended COLO 205 cells. Furthermore, flow cytometry results indicated that suspended cells express less surface E-cadherin and more cytoplasmic phosphorylated *β*-catenin compared to adherent cells (Figs. 3d and 3e).

### Manipulation of *β*-Catenin and E-cadherin Expression Result in Increased Number of Adherent Cells

**Figure 4:**
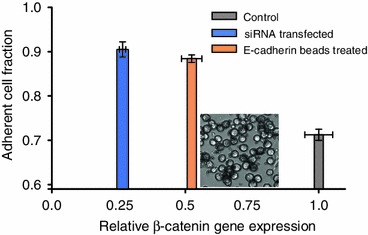
*β*-catenin gene expression was reduced after treating the overall COLO 205 population with siRNA. A 20% increase in the adherent cell fraction was observed. In a separate study, the overall COLO 205 cell population was incubated with E-cadherin antibody coated microspheres, which induced an increase in the adherent cell fraction and a 50% decrease in *β*-catenin gene expression

An average 75% decrease in *β*-catenin gene expression as measured by qPCR was achieved by treating COLO 205 cells with *β*-catenin siRNA. As a result of the significant decrease in *β*-catenin gene expression, the equilibrium ratio between adherent and suspended cells (7:3) shifted towards the adherent state, to a measured ratio of 9:1 (Fig. 4). This increase in the COLO 205 adherent cell population suggests that *β*-catenin may play a role in its own gene regulation, in addition to being an important linker protein in E-cadherin mediated cell–cell interactions. It was observed that the *β*-catenin siRNA was not able to completely abolish *β*-catenin mRNA expression, leaving enough *β*-catenin and reduced Wnt signaling to assist the *β*-catenin:E-cadherin complex mediated homotypic cell aggregation.^13^,^18^ Likewise, incubating COLO 205 cells with E-cadherin antibody coated microspheres, thereby externally increasing the probability of cell–cell adhesion mediated by E-cadherin expressed on the cells, was also found to induce a significant increase in the adherent cell fraction, shifting the ratio to 9:1 (Fig. 4). Furthermore, *β*-catenin gene expression decreased by 45% in these artificially clustered cells compared to control.

### Suspended COLO 205 Cells Express More Sialyl Lewis Acids and Show Significantly Stronger Interaction with E-selectin Coated Surfaces Under Physiological Shear Stress

**Figure 5:**
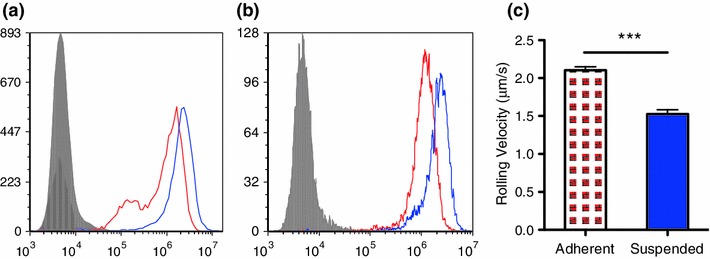
(a) and (b) Flow cytometry histogram plots of COLO 205 adherent (red) and suspended (blue) cells labeled with anti-sLe^x^ and anti-sLe^a^ antibodies. Isotype controls are shown in grey. (c) Rolling velocity analysis of adherent (red) and suspended (blue) under 1 dyn/cm^2^ shear stress. ***p < 0.0001

To further explore the population transition in COLO 205 cells, a flow based adhesion assay was used to investigate the adhesion phenotypes of both adherent and suspended populations. As shown in Fig. 5c, suspended COLO 205 cells were found to have a rolling velocity of 1.52 ± 0.05 *μ*m/s under 1 dyn/cm^2^ shear stress, significantly slower than the adherent cells rolling at 2.10 ± 0.04 *μ*m/s, indicating a more adhesive phenotype on the E-selectin coated surfaces. Compared to adherent cells, suspended cells were found to have elevated sLe^x^ and sLe^a^ expression by 60 and 80%, respectively (Figs. 5a and 5b).

### Human Plasma Induces a Pro-suspension Population Shift and Increased *β*-Catenin Expression

**Figure 6:**
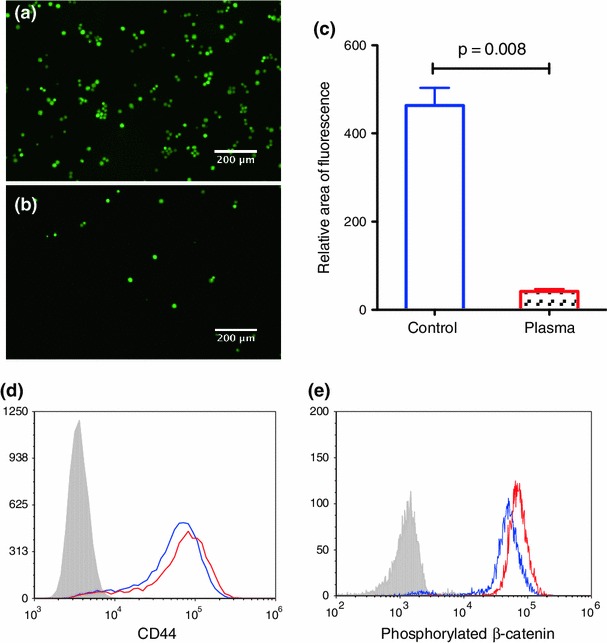
(a) and (b) Immunofluorescence images of the adherent cells after culturing in control and 50% plasma supplemented media, respectively. Cells were pre-incubated with fluorescently conjugated CellTracker dye prior to plating. (c) Quantification of the relative area of fluorescence with Image J. Two-tailed Student t-test was performed for statistical analysis (*p* = 0.008). (d) and (e) Flow cytometry histogram plots of CD44 expression on cell surfaces and intracellular phosphorylated *β*-catenin expression on the control (blue) and plasma treated (red), respectively. Isotype control was also performed and shown in grey

To assess whether COLO 205 cells may behave differently once they have entered the bloodstream, human plasma was isolated from healthy donors and added to the culture media. Interestingly, as shown in Figs. 6a and 6b, the majority of COLO 205 cells shifted to the suspended state, leaving significantly fewer adherent cells compared to control conditions with area of fluorescence quantified in Fig. 6c. Plasma-treated COLO 205 cells were also found to have greater expression of CD44, an E-selectin ligand, and phosphorylated *β*-catenin (Figs. 6d and 6e).

### Suspended Cells Show Increased Expression of Several EMT-Associated Genes

**Figure 7:**
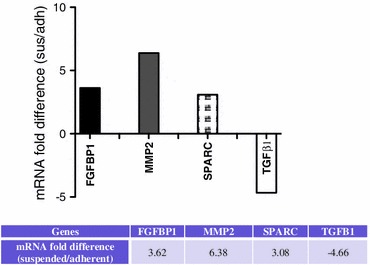
Real-time PCR of EMT-associated gene expression was performed in both suspended and adherent populations. mRNA expressions with >3-fold change include FGFBP1, MMP2, SPARC and TGF-*β*1. The fold difference is shown in both graph and table below after normalizing to expression level in the adherent cells

The dynamic switch between the two subpopulations of COLO 205 cells, with the adherent subpopulation showing increased expression of E-cadherin, and loss of E-cadherin expression in the suspended population combined with high expression of *β*-catenin and stronger interaction with E-selectin, is associated with the epithelial–mesenchymal transition (EMT), which is characterized by a loss of cell adhesion and increased cell invasion in many cancers. To examine this further, we assayed the expression of several EMT-associated genes in the adherent and suspended subpopulations using qPCR. Several EMT-associated genes were found to be upregulated in the suspended cells. Notably, mRNA of fibroblast growth factor binding protein 1 (FGFBP1), matrix metalloproteinase-2 (MMP2) and secreted protein that is acidic and rich in cysteine (SPARC) were >threefold higher in suspended COLO 205 cells, as shown in Fig. 7.

## Discussion

The heterogeneity of cancer is known to play an important role in determining the fate of cancer cells during the metastatic cascade. In this study, we characterized heterogeneity in the COLO 205 cancer cell line. This particular cell line has a subpopulation of cells that are adherent and another subpopulation of cells that are in suspended form. We showed that there is a dynamic switch between the two subpopulations of COLO 205 cancer cell lines and we further characterized the molecular heterogeneity in these two subpopulations.

The expression levels of phosphorylated kinases show that many kinases and their protein substrates are relatively more active in the suspended cell population compared to the adherent population. This is consistent with previous studies which suggest that the phosphorylation of proteins correlates with the mesenchymal phenotype of tumor cells.^7^,^24^ Two of the widely studied markers of epithelial and mesenchymal phenotypes are E-cadherin and *β*-catenin, respectively.^4^ Flow cytometry results reveal that adherent cells express more E-cadherin, while suspended cells express more phosphorylated *β*-catenin.

Manipulation of the *β*-catenin gene and surface E-cadherin expression in COLO 205 cells resulted in a shift in the population ratio with an increased number of adherent cells. Transfecting siRNA against *β*-catenin in COLO 205 cells shifted the adherent:suspended equilibrium ratio from 7:3 to ~9:1. Similarly, by introducing E-cadherin monoclonal antibody coated microbeads to the cell culture, the equilibrium ratio was also altered to ~9:1 and *β*-catenin gene expression was found to decrease by 50%. Taken together, these results suggest an interesting concurrent ‘inside-out’ and ‘outside-in’ regulatory system, where the decrease in *β*-catenin gene expression within these cells as well as the externally induced elevation of E-cadherin surface expression are both able to drive an increase in the adherent:suspended population ratio.

Interestingly, suspended COLO 205 cells were also found to roll on E-selectin at a significantly slower velocity under physiologically relevant shear stresses when compared to the adherent cell population. This suggests that if given the chance to intravasate into the blood vessel, the suspended cell population may establish stronger interactions with the inflamed endothelium. Recent studies from our group reported that blood plasma triggers an adhesive phenotypic switch of breast cancer cells on E-selectin coated surfaces under flow by upregulating E-selectin ligand and glycan expression.^5^,^8^ In this study, similarly, plasma treatment was found to elevate CD44 expression for COLO 205 cells, in addition to inducing a preferential shift from the adherent to the suspended cells, suggesting a more invasive phenotype with stronger interaction with the inflamed endothelium. Furthermore, upregulation of phosphorylated *β*-catenin expression was also observed. The dynamic population switch observed in this study suggests a potential mechanism, which increases the likelihood of extravasation of the circulating tumor cells from the bloodstream to develop secondary tumor sites. EMT is an essential process in the metastatic cascade but there are theories that suggest that EMT is a continuous process that could happen while the cancer cells are in the bloodstream. EMT markers are reported to be expressed by CTCs captured from breast cancer patients indicating that factors in blood could aid in the EMT process.^32^ Plasma is rich in pro-inflammatory cytokines^8^ that aid in the metastatic process and we found an interesting switch of COLO 205 cells preferentially to the suspended state showing increased expression of E-selectin ligands when treated with human plasma.

Several EMT-associated genes including FGFBP1, MMP2 and SPARC are upregulated in suspended COLO 205 cells. FGFBP1 and MMP2 have been previously shown to be upregulated in metastatic colorectal cancers relative to normal colon epithelia.^23^,^30^ As a mesenchymal cell marker, SPARC expression has been identified during breast cancer EMT which correlates with a basal-like phenotype.^25^ In contrast, transforming growth factor beta 1 (TGFB1) was downregulated in the suspended population of COLO 205 cells compared to the adherent population. As TGFB1 is considered to drive EMT through both paracrine and autocrine signaling,^26^ its transient upregulation in the adherent COLO 205 population may induce EMT and thus promote the transition to the suspended state. These results strongly suggest that the transition from the adherent to the suspended state creates cells that express both mesenchymal-like phenotype and genotype from a cell line of epithelial origin and may be considered EMT-like.

The suspended population expressed significantly more phosphorylated *β*-catenin and less E-cadherin compared to the adherent population. In epithelial cells, the intracellular domain of surface E-cadherin is associated with *β*-catenin. When a cell undergoes EMT there is a loss of E-cadherin expression and increased translocation of *β*-catenin to the nucleus. “Inside-out” manipulation *via* *β*-catenin siRNA knockdown assay to reduce the expression of cytoplasmic *β*-catenin, and “outside-in” manipulation *via* introduction of E-cadherin antibody coated microspheres to increase E-cadherin mediated cell–cell adhesion, shifted the equilibrium population ratio to 9:1. Therefore, we conclude that the expression of *β*-catenin and E-cadherin regulate the dynamic switch of COLO 205 cells as two distinct subpopulations.

In the context of metastasis, the suspended COLO 205 population represents a more invasive phenotype. Suspended cells expressed 60% more sLe^x^ and 80% more sLe^a^ than adherent cells, which resulted in significantly slower, rolling velocities on E-selectin coated microtubes. Moreover when cells were cultured in human plasma, the expression of phosphorylated *β*-catenin increased, shifting the majority of cells into the suspended population where sLe^x^ and E-selectin ligand CD44 expression both increased as well. Upregulation of the EMT markers FGFBP1, MMP2 and SPARC within the suspended population suggests mesenchymal-like cells and therefore a much more aggressive population. Future studies could focus on the regulation of phosphorylated *β*-catenin or E-cadherin to control the aggressiveness of colorectal cancers to help prevent metastases.
